# The impact of patient choice on uptake, adherence, and outcomes across depression, anxiety, and eating disorders: a systematic review and meta-analysis

**DOI:** 10.1017/S0033291725000066

**Published:** 2025-02-07

**Authors:** Catherine Johnson, Marcela Radunz, Jake Linardon, Matthew Fuller-Tyszkiewicz, Paul Williamson, Tracey D. Wade

**Affiliations:** 1 Flinders University Institute of Mental Health and Wellbeing and Blackbird Institute, Adelaide, Australia; 2 SEED Lifespan Strategic Research Centre, School of Psychology, Faculty of Health, Deakin University, Geelong, Australia; 3 Office of the Executive Dean, Faculty of Health, Deakin University, Geelong, Australia

**Keywords:** choice, eating disorders, mental health, meta-analysis, preference, review

## Abstract

Growing evidence highlights the critical role of patient choice of treatment, with significant benefits for outcomes found in some studies. While four meta-analyses have previously examined the association between treatment choice and outcomes in mental health, robust conclusions have been limited by the inclusion of studies with biased preference trial designs. The current systematic review included 30 studies across three common and frequently comorbid mental health disorders (depression *N* = 23; anxiety, *N* = 5; eating disorders, *N* = 2) including 7055 participants (*M*_age_ 42.5 years, SD 11.7; 69.5% female). Treatment choice most often occurred between psychotherapy and antidepressant medication (43.3%), followed by choice between two different forms of psychotherapy, or elements within psychotherapy (36.7%). There were insufficient studies with stringent designs to conduct meta-analyses for anxiety or eating disorders as outcomes, or for treatment uptake. Treatment choice significantly improved outcomes for depression (*d* = 0.17, *n* = 18) and decreased therapy dropout, both in a combined sample targeting depression (*n* = 12), anxiety (*n* = 4) and eating disorders (*n* = 1; OR = 1.46, 95% CI: 1.17, 1.83), and in a smaller sample of the depression studies alone (OR = 1.65, 95% CI: 1.05, 2.59). All studies evaluated the impact of adults making treatment choices with none examining the effect of choice in adolescents. Clear directions in future research are indicated, in terms of designing studies that can adequately test the treatment choice and outcome association in anxiety and eating disorder treatment, and in youth.

## Introduction

The use of mental health services is escalating. Utilization and spending rates for mental health care services among commercially insured adults in the USA increased by 38.8% and 53.7%, respectively, from 2019 to 2022 (Cantor et al., 2023). At the same time, various indicators show that mental health is deteriorating at a population level. The Australian National Study of Mental Health and Wellbeing (Australian Bureau of Statistics, 2022) revealed that the prevalence of operationally defined mental disorders in 16–24-year-olds rose from 26% in 2007 to 40% in 2021. Globally, 27% of responders across 64 countries in 2022 were classified as distressed or struggling with mental health, representing a decline of 11% between 2019–2021, with no recovery towards pre-pandemic levels evident (Sapien Labs, 2023).

While potential solutions are varied and complex, one approach seeks to identify ways in which we can optimize our existing evidence-based interventions. For example, personalizing treatment to the individual (e.g., tailoring treatment to a unique symptom profile measured at baseline, or using machine learning to identify patient subgroups) demonstrates better outcomes (small but significant) relative to a “one-size-fits-all” approach (Nye, Delgadillo, & Barkham, 2023). Adding further significant benefit to the use of a purely clinician-tailored treatment is the incorporation of some degree of client choice regarding therapy (Andersson et al., 2023). Understanding the contribution of different optimization approaches and how best to apply these has the potential to translate to a sizeable increase in the number of clients meeting remission after treatment, thus improving the cost-effectiveness of mental health treatments.

While robust meta-analyses have emerged pertaining to different optimization approaches (e.g., de Jong et al., 2021; Nye, Delgadillo, & Barkham, 2023), outcomes related to the utilization of client treatment choice are less certain. To date, four systematic reviews with meta-analyses have investigated the impact of patient preference (Table 1). Two included both medical and psychological conditions (Delevry & Le, 2019; Lindhiem, Bennett, Trentacosta, & McLear, 2014), and two focused on mental health (Swift, Callahan, Cooper, & Parkin, 2018; Windle et al., 2020). The latter two studies showed a benefit of patient preference on reduced dropout rates (OR 1.79; RR 0.62 respectively). One demonstrated superior clinical outcomes (*n* = 53; *d* = 0.28; Swift, Callahan, Cooper, & Parkin, 2018) moderated by diagnosis (depression/anxiety > psychosis/behavioral/substance use disorders), but one found no effect on depression, anxiety, or global outcomes (*n* = 29; Windle et al., 2020).

**Table 1.** Overview of systematic reviews and meta-analyses investigating patient preference

Author (year)	Conditions included (N)	Study designs	Age targeted	Impact of choice/preference matching	Moderators
Lindhiem, Bennett, Trentacosta, and McLear (2014)	Depression (12) Medical (11) Other (9)	Preference design = any; RCTs	Any; Age across included = NR	Greater satisfaction (*d* = .34) Increased completion rates (OR = 1.37) Superior clinical outcomes (*d* = .15)	Diagnostic groups (depression vs medical) ns
Windle et al. (2020)	Mental health (29)	Preference design = any; RCTs	Adult	Reduced dropout (RR = .62) Greater therapeutic alliance (*d* = .48) Global outcomes ns Depression/anxiety outcomes ns Satisfaction ns	Age ns Gender ns Race ns
Swift, Callahan, Cooper, and Parkin (2018)	Mental health (53)	Preference design = any	Adult	Reduced dropout (OR = 1.79) Superior clinical outcomes (*d* = .28)	Study design on dropout and outcomes (correlational, RCT > PRPT, choice/no-choice) Diagnosis on outcomes (Anx/Dep > behavioral, psychosis, substance use, other) Preference type (treatment, activity, therapist), age, gender, ethnicity, years of education all ns
Delevry and Li (2019)	Mental health (9)^a^ Pain/functional disorders (14) Weight loss (4)^b^	Restricted to FRPT (21) and DRPT (6); RCTs	Any; one study recruited from age 12^c^	Superior clinical outcomes (*d* = .18)	Mental health>pain/functional DRPT>FRPT

a Of these nine mental health studies, three were DRPT design, six were FRPT; eight trials compared psychotherapy to pharmacotherapy, one compared psychological approaches.

b This group excluded from results as contributed to substantial heterogeneity.

c One included study recruited from age 12 in a trial comparing medical approaches (*M*_age_ of the study = 23.7 years).

These mixed results are likely explained by the inclusion of three differing preference trial designs, as described by Delevry and Le (2019). The first are *Partially Randomised Preference Trials* (PRPTs) where those who decline randomization choose their preferred treatment and continue in a trial. While this design retains more participants, it does not allow an unbiased measure of the impact of treatment choice. Second, a *Fully Randomised Preference Trial* (FRPT) assesses patient preference at baseline, but all participants proceed to randomization, allowing comparison of those who are matched versus mismatched to their preferred treatment. However, mismatch may be biased by “resentful demoralization.” Third, and by contrast, a *Doubly Randomised Preference Trial* (DRPT) randomizes participants to a choice vs no-choice arm, before further randomizing to Treatment A or B within the no-choice arm, and is thus the only design allowing true estimation of the impact of active choice. The FRPT design is more common given it is easily accommodated in a standard Randomised Controlled Trial (RCT) through the addition of a simple preference question at baseline. Delevry and Le (2019) were the first to exclude PRPT designs in the most recent of the four meta-analyses in a mixed sample across mental health (*n* = 9) and pain/functional disorders (*n* = 14), showing client preference matching conferred superior clinical outcomes (*d* = 0.18). The effect was stronger for mental health treatments (*d* = 0.23) and when limiting studies to more stringent DRPT designs (*d* = 0.27).

Given only nine studies of mental health preference were included in the most stringent meta-analysis, we searched the literature for more recent studies. In particular, we ensured our search terms could detect studies of eating disorders, a notable omission in the extant meta-analyses given these are a common, costly public health concern among youth and young adults, affecting one in six females and one in 40 males (Silén et al., 2020). Only 35.4% of clients completing treatment for bulimia nervosa experience remission (Linardon & Wade, 2018), so understanding how client choice might enhance outcomes is of vital importance. We also wished to search for preference studies in youth, as none to date have been included in meta-analyses. Swift, Callahan, Cooper, and Parkin (2018) described the difficulty of including children and adolescents in preference trials, where it is more difficult to determine whose preference is driving treatment decisions. However, choice may be particularly powerful in adolescent samples, given the developmental push for autonomy that emerges at this age (e.g., Johnson, Taylor, Dray, & Dunning, 2024).

The primary aim of the current study was therefore to conduct an updated meta-analysis of the impact of patient choice on treatment outcomes across three common and frequently comorbid mental health disorders: anxiety, depression, and eating disorders. We included any age group to determine if studies have emerged in youth. At the systematic review stage, we maintained a broad net (i.e., allowing any study design) to identify emerging research on eating disorders and/or in youth, neither of which have been reported in existing preference reviews. For meta-analyses, we followed the recommendations of Delevry and Le (2019) to focus on robust preference trial designs (i.e., excluding PRPTs). Finally, we also sought to conduct the first meta-analysis of treatment uptake.

## Method

### Search strategy and selection criteria

This review was conducted according to the PRISMA guidelines (Page, McKenzie, et al., 2021; Page, Moher, et al., 2021). The following search string was used: ((choice or prefer*) and (treatment or interv* or trial or therapy or self-help or program* or e-health) and random*) in the title only and (depress* or anxi* or eating* or bulimi* or anorexi* or psychia*) in the abstract.

Inclusion criteria were as follows: (1) Peer-reviewed (published) journal articles; (2) RCTs where at least one treatment arm offered patients a choice of approach for eating disorders, depression, and/or anxiety; (3) outpatient setting; (4) quantitative mental health outcomes reported for patient preference-matched or choice versus preference mismatched or no choice groups; (5) any age range; (6) any publication year; (7) any language. Studies were excluded if they met the following criteria (1) Reviews, meta-analyses, or protocols; (2) secondary analyses with no additional extractable data related to patient preference. Studies that met the first exclusion criterion were tagged for reference list searching, however.

The study protocol was registered on the Open Science Framework (https://doi.org/10.17605/OSF.IO/SNJXR) on April 8, 2024, and searches of three electronic databases Medline, PsycINFO, and Scopus, were conducted on April 11, 2024. An updated search was performed on December 5, 2024, including the addition of grey literature via the database ProQuest dissertations & theses global, to more fully explore publication bias. Results of the search were loaded into Covidence software where duplicates were removed, and then the title and abstracts were double-screened by two independent reviewers (CJ and MR) before the same authors independently examined the full text of the surviving articles. Inter-rater reliability was very good at both stages (Cohen’s Kappa = .84, .93 respectively). Conflicts were resolved by discussion between reviewers.

### Data extraction

One of two authors (CJ, MR) extracted the following information for each included study: author, year, country, sample size, participant demographics (mean age, gender, ethnicity, and socioeconomic status), mental health disorder targeted (eating disorder, depression or anxiety), study design (partial, fully or doubly randomized preference trial), intervention arm comparators (e.g., antidepressant medication versus psychotherapy) and whether the strength of preference was assessed. The following data were extracted for meta-analyses: mean and standard deviation of mental health outcomes at baseline and end of treatment in *choice/matched preference* versus *no-choice/non-matched preference* groups to calculate effect sizes (Cohen’s *d*). Frequency/percentage of uptake (i.e., participants that attended the initial session) and dropout rates (i.e., participants who did not complete treatment, or did not complete a sufficient dose if defined *a priori* by study) were also extracted for both groups to calculate odds ratios. Where raw data was not available for these syntheses, we used an online effect size calculator (https://www.campbellcollaboration.org/calculator/) to convert reported results; where insufficient data were reported we emailed authors for raw data if papers were < 15 years old; where no data were extractable through any approach described, we report narrative descriptors of preference effect from study text. Where more than one outcome was reported for a mental health construct (e.g., depression), the following sequence was applied: the primary outcome was extracted if nominated (if two were nominated both were extracted given multilevel approach adjusts for a unit of analysis error); then a clinician-reported outcome if available; then self-report outcome most relevant to target condition (e.g., social anxiety rather than generalized anxiety for a study targeting social anxiety disorder). If a study reported two different outcomes (e.g., targeted anxiety but also measured depression) both were extracted. Where low study numbers precluded meta-analyses, we extracted descriptors of preference effects from study text for narrative syntheses. Where these were not reported but data were provided, we manually calculated outcome (Cohen’s *d*) or engagement effects (Odds ratios) for single studies to include a result (i.e., choice or preference did or did not improve outcome) in our descriptive syntheses.

### Quality assessment

Quality assessment utilized a subset of items from the 25-item Consolidated Standards of Reporting Trials (CONSORT) checklist (Schulz et al., 2010). Ten criteria of most relevance to included studies (i.e., RCTs) were selected by the study team as follows: Item 4a: eligibility criteria for participants/recruitment method; Item 4b: settings and locations where the data were collected/care providers described; Item 5: Description of interventions with sufficient detail to allow replication; Item 6a: use of validated scales with properties reported and primary outcome(s) nominated; Item 7: how sample size was determined; Item 8a: method used to generate randomization reported; Item 12a: statistical methods described in sufficient detail, with treatment effects including 95% confidence intervals plus reporting of ITT; Item 13a: the numbers of participants who were assessed for eligibility, randomly assigned, received intended treatment and were analyzed for the primary outcome; Item 13b: reasons for losses and exclusions; Item 15: a table showing baseline demographic and clinical characteristics. Items were scored ‘Y’ when completely conforming with the criteria, ‘N’ when not fully conforming with the criteria, and ‘P’ when partially conforming to the CONSORT criteria. Overall, studies were rated as high quality if they fully conformed with 8 of 10 criteria.

### Meta-analyses

All meta-analyses were conducted with the *R* statistical software program, using the *metafor* package. Following Cochrane’s recommendations (Higgins & Green, 2011) we required each outcome to have a minimum of 10 studies to proceed with a rigorous meta-analysis. Meta-analyses were conducted by comparing outcomes for participants offered choice or matched to preference to those who were randomized or non-matched to preference. For each group separately, we first calculated the mean gain (such that a positive score indicated improvement) and standard deviation of the difference. Between-group effect sizes were then precalculated from these scores using the Campbell Collaboration effect size calculator (https://www.campbellcollaboration.org/calculator/). Where a study reported preference data separately by treatment arm (e.g., match vs mismatch for antidepressant therapy, and for psychological therapy) both were extracted and entered into the analysis separately; we used a multi-level approach to account for non-independence of effects (Harrer, Cuijpers, Furukawa, & Ebert, 2021). Binary outcomes (i.e., odds ratios for uptake and dropout) were manually converted to log odds ratios for the multilevel analyses and exponentiated back to odds ratios for ease of interpretation in tables and forest plots (Harrer, Cuijpers, Furukawa, & Ebert, 2021). PRPT designs were not included in meta-analyses. Meta-analyses were planned with both double and fully randomized trials for maximum power, and then just with the former design as a more stringent test of choice effect. Heterogeneity was assessed with a combination of the *Q* and I^2^ statistics, and Egger’s regression test was used as one indicator of publication bias (Egger, Smith, Schneider, & Minder, 1997), entered as a moderator in our multilevel models.

## Results

### Description of included studies

**Figure 1.**
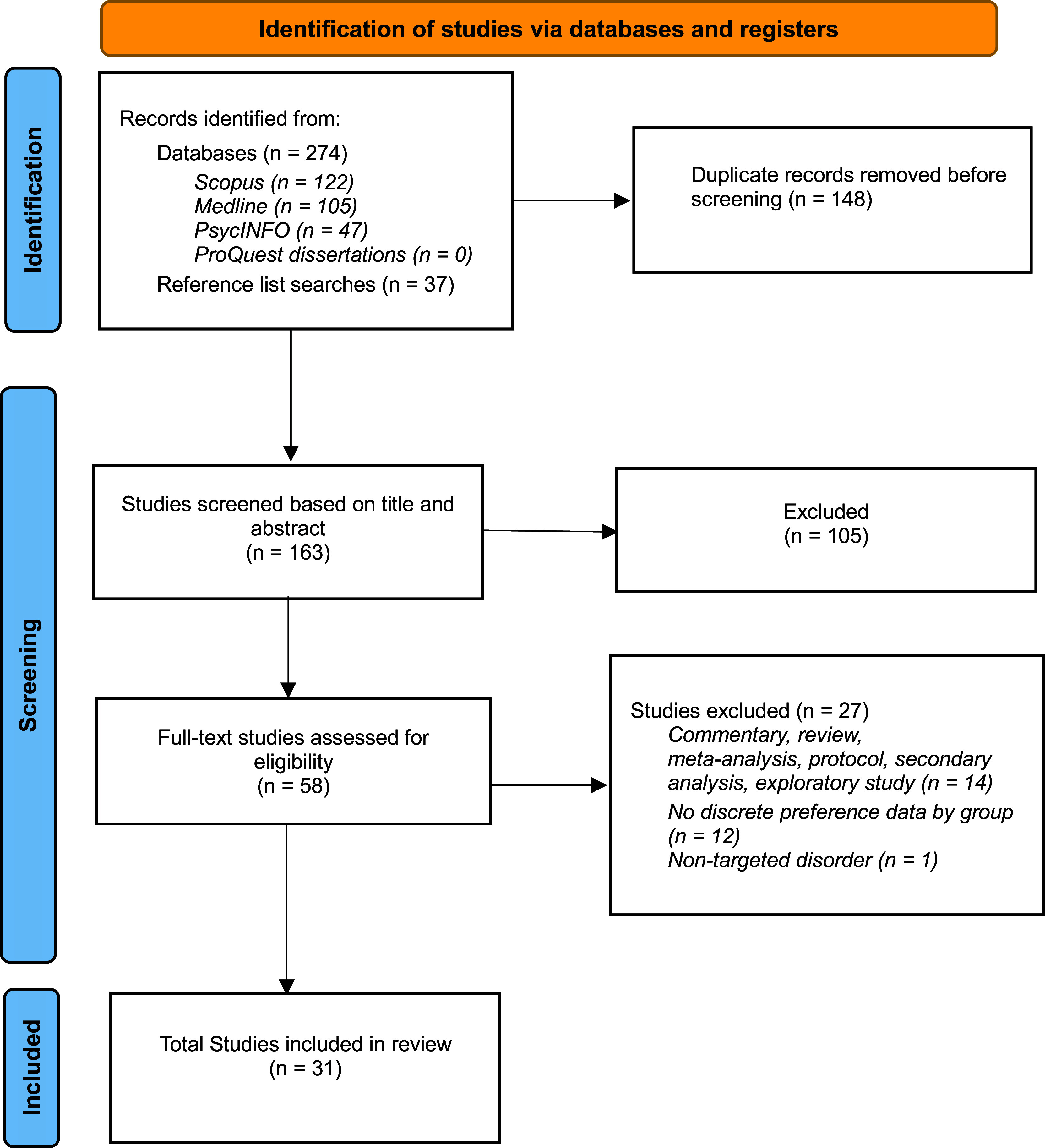
PRISMA diagram of study selection process.

The search yielded 311 studies; after removing duplicates, 163 remained (Figure 1). No unpublished studies were identified. Title and abstract screening excluded 105 studies, with 58 proceeding to full text screening where a further 27 were excluded. A full list of exclusion reasons appears in Supplementary Table S1.

The final sample for the systematic review consisted of 31 papers reporting 30 studies across 7055 participants (*M*_age_ 42.5 years, SD 11.7; 69.5% female; Table 2). Of these, one secondary paper contributed an additional outcome measure. Most studies targeted depression (*n* = 23; 76.7%), with anxiety represented in 5 studies (16.7%) including its variants panic disorder, social anxiety, and obsessive-compulsive disorder. Only two studies (6.7%) targeted eating disorders (binge eating disorder and prodromal anorexia nervosa). Most frequently, the choice offered to patients was between antidepressant medication and psychotherapy (*n* = 13; 43.3%) or between two types of psychotherapy (*n* = 11; 36.7%). Most studies (*n* = 15; 50.0%) were conducted in the USA, followed by Europe and the UK (*n* = 5; 16.7% each). Smaller numbers of studies were from Scandinavia (*n* = 2; 0.07%), Australia, Canada, and Iran (*n* = 1; 0.03% each).

**Table 2.** Study characteristics

Author (Year)	Demographics (N Whole Sample; Mean Age (SD); % female, Ethnicity, SES)	Mental health condition	Study design	Comparison category	Outcome measure extracted
Andersson et al. (2023) Sweden^a^	N = 197; Mage = 34.6 (SD 13.2); 77.2% Female; Ethnicity = NR; SES = university education 75.7%	Depression	DRPT	Psych vs Psych (self vs clinician tailored)	Anxiety (Narrative); Depression (BDI–2, PHQ–9 = two primary outcomes); QoL (Narrative); Dropout rates
Bakker et al. (2000) Netherlands	N = 66; Mage = 33.9 (SD 8.3); 74% Female; Ethnicity = NR; SES=NR	Panic disorder	PRPT	Psych vs Psych (CT, randomized vs preference)	Anxiety (Narrative); Depression (Narrative)
Bedi et al. (2000) UK	N = 323; Mage = 37.4 (SD 11.4); 75% Female; Ethnicity = NR; SES = 34% Professional/managerial; 38% Skilled; 29% Partly/Unskilled	Depression	PRPT	Psych (counseling) vs ADM	Depression (Narrative); Uptake rates (Narrative)
Brenes et al. (2020) USA^a^	N = 500; Mage = 66.5 (SD 5.2); 86.6% Female; Ethnicity 78.8% White, 14.8% Black, 6.4% other; SES = 54.6% College education	Anxiety	DRPT	Psych (CBT) vs other (Yoga)	Anxiety (Narrative); Uptake (Narrative) and Dropout rates
Danhauer et al. (2022) secondary paper to Brenes et al. (2020)^a^					Depression (PROMIS–29)
Callaghan, Khalil, Morres, and Carter (2011) UK^a^	N = 38; Mage = 53.7 (SD 12.6); 100% Female; Ethnicity = NR; SES=NR	Depression	DRPT	Other (prescribed vs preferred exercise)	Depression (BDI); QoL (Narrative); Dropout rates
Cooper et al. (2018) UK^a^	N = 36; Mage = 44.0 (SD 17.8); 83.3% Female; Ethnicity 97.2% White, 2.8% Asian; SES = 25% College education	Depression	FRPT	Psych (Person-centred) vs Psych (CBT GSH)	Depression (PHQ–9); QoL (Narrative); Uptake rates (Narrative)
Dowrick et al. (2011) UK^a^	N = 220; Mage = 39.9 (SD NR); 70.0% Female; Ethnicity 89% White; SES = Mage completed education 17.7 (SD = 3.9)	Depression	FRPT	Psych (supportive care from GP) alone vs Psych + ADM	Depression (HDRS); Dropout rates
Dunlop et al. (2012) USA^a^	N = 80; Mage = 41.6 (SD 8.3); 57% Female; Ethnicity 71% White, 22% Black, 8% other; SES=NR	Depression	FRPT	Psych vs ADM	Depression (HDRS); Anxiety (Narrative); Dropout rates
Dunlop et al. (2017) USA^a^	N = 344; Mage = 40 (SD 11.7); 57% Female; Ethnicity 47.7% White, 18.6% Black, 33.7% other, 29.7% Hispanic, 70.3% Non-Hispanic; SES = 69.5% some College education	Depression	FRPT	Psych vs ADM (2 medication types)	Depression (HDRS)
Elkin et al. (1999) USA^a^	N = 82; Mage = NR; Gender = NR; Ethnicity = NR; SES=NR	Depression	FRPT	Psych (Combined CBT, IPT) vs ADM	Depression (BDI); Dropout rates
Gum et al. (2006) USA	N = 1602; Mage = 70.0 (SD NR); 67% Female; Ethnicity: 77% White, 12% Black, 8% Hispanic, 3% Other; SES = 81% > High School Graduate.	Depression	FRPT	Other: TAU vs collaborative care	Depression (Narrative)
Hegerl et al. (2010) Germany^a^	N = 368; Mage = 46.4 (SD 14.6); 68.2% Female; Ethnicity = NR; SES=NR	Depression	DRPT	Other: Allocated to CBT, GSH, ADM, placebo vs choice arm	Depression (Narrative); Uptake (Narrative); Dropout rates
Kocsis et al. (2009) USA^a^	N = 429; Mage = 45.0 (SD NR); 65% Female; Ethnicity 92% White; SES=NR	Depression	FRPT	Psych (CBT) vs ADM	Depression (HDRS); Dropout rates
Koszycki, Ilton, Dowell, and Bradwejn (2022) Canada^a^	N = 97; Mage = 40.4 (SD 13.7); 62.3% Female; Ethnicity 79.6% White; SES = 70.5% College education	Social Anxiety Disorder	FRPT	Psych (CBT) vs Psych (MBI)	Anxiety (Narrative); Depression (BDI–2); QoL (Narrative); Uptake (Narrative); Dropout rates
Kwan, Dimidjian, and Rizvi (2010) USA^a^	N = 106; Mage = 38.4 (SD 11.7); 64.2% Female; Ethnicity 79.2% White, 3.8% Black, 6.6% Hispanic/Latino, 10.3% other; SES = 63.2% College education	Depression	FRPT	Psych (CBT) vs Psych (BA)	Depression (Narrative); Dropout rates
Leuzinger-Bohleber et al. (2019) Germany	N = 252; Mage = 40.62 (SD NR); 67.5% Female; Ethnicity = NR; SES = 65.9% High School Graduate	Depression	PRPT	Psych (CBT) vs Psych (Psychodynamic)	Depression (Narrative)
Leykin et al. (2007) USA^a^	N = 240; Mage = 40.0 (SD 12.0); 59% Female; Ethnicity 81.3% White; SES = predominantly some College education	Depression	FRPT	Psych (CT) vs ADM	Depression (HDRS); Dropout rates
Lin et al. (2005) USA^a^	N = 335; Mage = 57.0 (range = 24–84); 5% Female; Ethnicity 78.8% White; SES=NR	Depression	FRPT	Other: TAU vs collaborative care	Depression (HSC–90)
Linardon et al. (2023) Australia^a^	N = 155; Mage = 34.1 (SD = 10.56); 94.8% Female; Ethnicity 88.3% = White, 2.6% = Mixed, 3.9% = Asian, 1.3% = Black, 3.9% = Other; SES = 92.2% > High School Graduate	Binge Eating Disorder	DRPT	Psych (Target: Inflexible dieting, mood intolerance & body image concerns) vs Psych (Target: Inflexible dieting)	Disordered Eating (Narrative); Uptake (Narrative); Dropout rates
Loeb et al. (2020) USA	N = 59; Mage = 13.70 (SD 2.09); Gender = 84.7%; Ethnicity 84.7% = White, 10.2% = Hispanic/Latino, 3.4% = Asian; SES=NR	Anorexia Nervosa (prodromal)	PRPT	Psych (FBT) vs Psych (Supportive Psychotherapy)	Disordered Eating (Narrative)
Mergl et al. (2011) Germany^a^	N = 117; Mage = 43.4 (SD 13.1); 65.9% Female; Ethnicity = NR; SES=NR	Depression	DRPT	Psych (CBT) vs ADM	Depression (HDRS); Dropout rates
Moradveisi, Huibers, Renner, and Arntz (2014) Iran	N = 100; Mage = 31.4 (SD 9.0); 85% Female; Ethnicity = NR; SES=NR	Depression	FRPT	Psych (BA) vs ADM	Depression (Narrative)
Raue et al. (2009) USA^a^	N = 60; Mage = 51.2 (SD 17.4); 78% Female; Ethnicity 33% White non-Hispanic, 32% White and Hispanic, 8% Black and Hispanic, 2% African American, 2% Asian, 5% other; SES = 15% < High School Graduate	Depression	DRPT	Psych (IPT) vs ADM	Depression (HDRS); Uptake rates (Narrative)
Rokke, Tomhave, and Jocic (1999) USA^a^	N = 40; Mage = 66 (SD 6.1): range: 60–86; 38% Female; Ethnicity = NR; SES=NR	Depression	DRPT	Psych (Target: Behaviour) vs Psych (Target: Cognitive)	Depression (HDRS); Dropout rates
Svensson et al. (2021) Sweden^a^	N = 221; Mage = 34.9 (SD 12.6); 75% Female; Ethnicity = NR; SES = 37% College Education	Panic disorder	DRPT	Psych (Psychodynamic) vs Psych (Panic control)	Anxiety (Narrative); Depression (MADRS); QoL (Narrative); Uptake (Narrative); Dropout rates
Uebelacker et al. (2018) USA^a^	N = 122; Mage = 46.5 (SD 12.1); 84% Female; Ethnicity 84% White, 12% Multiracial, 3% Black; SES 58% College education	Depression	FRPT	Other: Yoga vs Health education	Depression (QIDS)
Van et al. (2009) Netherlands^a^	N = 119, Mage = 35.9 (SD 10.4); 80% Female; Ethnicity NR; 30%/47%/23% = high/intermediate/low education level	Depression	PRPT	Psych (Psychodynamic) vs ADM	Depression (Narrative); Dropout rates
Ward et al. (2000) UK	N = 464; Mage = 37.0 (SD 12.2); 75% Female; Ethnicity 89.9% White; 64% Professional/managerial/skilled non-manual	Depression	PRPT	Psych (CBT) vs Psych (Non-directive counseling)	Depression (Narrative)
Wheaton et al. (2016) USA^a^	N = 80; Mage = 33.9 (SD 11.4); 48% Female; Ethnicity 90% White non-Hispanic; SES = 16.6 (2.5) Mean years of education	OCD	FRPT	Psych (Exposure) vs antipsychotic augmentation of ADM	Anxiety (Narrative); Dropout rates
Zlotnick, Elkin, and Shea (1998) USA^a^	N = 203; Mage = 35 (SD 8.5); 69.5% Female; Ethnicity White 89%; SES = 75% some College education	Depression	FRPT	Other: therapist gender preference	Depression (HDRS); Dropout rates

Abbreviations: ADM = antidepressant medication; BBQ = Brunnsviken Brief Quality of Life Scale; BDI=Beck Depression Inventory; DRPT = Doubly Randomised Preference Trial; DRPT = Doubly Randomised Preference Trial; EQ-5D = EuroQoL 5 Dimension scale; FRPT = Fully Randomised Preference Trial; FRPT = Fully Randomised Preference Trial; GAD-7 = Generalised Anxiety Disorder scale; HARS=Hamilton Anxiety Rating Scale; HDRS=Hamilton Depression Rating Scale; HSC-90 = Hopkins Symptoms Checklist; MADRS = Montgomery-Asberg Depression Rating Scale; PDSS=Panic disorder severity scale; PHQ = Patient Health Questionnaire; PRPT = Partially Randomised Preference Trial; PRPT = Partially Randomised Preference Trial; QIDS = Quick Inventory of Depression Symptomatology; QLDS = Quality of Life in Depression Scale; ROMIS=Patient-Reported Outcomes Measurement Information System; SAS=Social Anxiety Scale; SDS=Sheehan Disability Scale; SWLS=Satisfaction with Life Scale; YBOCS=Yale-Brown Obsessive Compulsive Scale.

a Denotes included in meta-analyses.

By preference study design, in decreasing order of rigor, 9 studies (30.0%) were doubly randomized (DRPT), 15 studies (50.0%) were fully randomized (FRPT), and 6 (20.0%) were partially randomized (PRPT). PRPT designs appear in Table 2, but were not included in our narrative or meta-analytic syntheses.

### Meta-analyses

**Mental health outcomes.** Follow-up data were reported in only 50% of studies, so post-intervention data were utilized. There were insufficient numbers of studies to allow meta-analyses for two of three mental health outcomes (anxiety and eating disorders) and insufficient DRPT designs for depression to run a more stringent analysis utilizing this design alone. One meta-analysis was therefore conducted, for depression, using a combined sample of DRPT and FRPT designs (Table 3), and including one study that targeted anxiety but also measured depression. This analysis showed a small significant effect (Cohen’s *d* = 0.17; 95% CI .08, .27; I^2^ = 13.55) such that depression was lower for those who chose or were matched to their preferred treatment. The Forest plot appears in Supplementary Figure 1. Egger’s test suggested no evidence of publication bias; we also found no unpublished studies in our search of the grey literature.

**Table 3.** Meta-analysis results for depression and dropout rates, by preference group

Outcome	*k* comparator arms (nested in *n* studies)	DRPT designs included *N* studies (%)	Effect size (95% CI)	*p*	Total *I^2^*	Cochran’s Q, *p*	Eggers’ test *p*
Depression	25 (18)	7 (38.9%)	.17 (.08,.27)^a^	<.001	13.55%	27.75, *p* = .27	.78
Dropout (mixed target conditions)^c^	22 (17)	8 (47.1%)	1.46 (1.17, 1.83)^b^	<.01	9.69%	29.03, *p* = .11	.13
Dropout (depression only)	13 (12)	5 (41.7%)	1.65 (1.05, 2.59)^b^	.03	47.68%	23.84, *p* = .02	.21

a Effect size = Cohen’s d.

b Effect size = Odds Ratio.

c Mixed group comprises depression = 13 arms/12 studies, anxiety = 7 arms /4 studies; eating disorders = 2 arms /1 study.

**Dropout and uptake rates.** There were insufficient numbers of studies to conduct a quantitative synthesis for uptake rates, or for dropout with DRPT designs alone; we ran the following two meta-analyses using a combined sample of DRPT and FRPT designs. First, dropout rates across a mixed group of target disorders for maximum power, and second, dropout rates for depression alone (Table 3).

Treatment choice had a significant effect on dropout rates, in both samples. In the larger combined sample (targeting depression, *n* = 12; anxiety, *n* = 4 and eating disorders, *n* = 1 study), participants who were not offered a choice, or were not matched to their preferred treatment, were 1.46 times (95% CI 1.17,1.83; I^2^ = 9.69%) more likely to drop out. In the subsample of 12 studies targeting depression alone, those randomized to treatment were 1.65 times (95% CI 1.05,2.59; I^2^ = 47.68%) more likely to drop out compared to those allowed to choose their treatment approach. However, we note the wider confidence interval and greater heterogeneity in the smaller sample. Combined with the absence of unpublished studies, Eggers’s test added no evidence of publication bias for either sample. Forest plots for dropout rates appear in Supplementary Figures S2–S3.

### Studies not included in meta-analyses

**Mental health outcomes. *Depression.*** Data were not able to be extracted for four studies (three FRPT, one DRPT) targeting depression to include in the above meta-analyses. Three found no benefit to preference matching (Gum et al., 2006, *N* = 1602; Kwan, Dimidjian, & Rizvi, 2010; *N =* 106) or choice (Hegerl et al., 2010, DRPT, *N* = 368). Moradveisi, Huibers, Renner, and Arntz (2014), *N =* 100) found preference scores predicted dropout from pharmacological but not psychological (behavioral activation) treatment; preference scores also positively impacted the initial response within the behavioral activation arm. Considering DRPT studies (*N* = 7 studies) as a separate group, three found a benefit for choice (Andersson et al., 2023, *N* = 197; Callaghan, Khalil, Morres, & Carter, 2011, *N* = 38; Mergl et al., 2011, *N* = 117) while four studies found no effect (Danhauer et al., 2022, *N* = 500; Raue et al., 2009, *N* = 60; Rokke, Tomhave, & Jocic, 1999, *N* = 40; Svensson et al., 2021, *N* = 221). No pattern of the type of choice (e.g., the choice between two types of psychotherapy, or between medication and psychotherapy) driving benefit or lack thereof was evident between the groups.

***Anxiety.*** In the three studies that utilized a DRPT design (Andersson et al., 2023, *N* = 197; Brenes et al., 2020, *N* = 500; Svensson et al., 2021, *N* = 221), no benefit of offering choice was found. Of three FRPT studies, two found no impact of preference matching (Dunlop et al., 2012, *N* = 80; Koszycki, Ilton, Dowell, & Bradwejn, 2022; *N* = 97) while one (Wheaton et al., 2016; *N* = 80) found the effect differed by condition: matching did not affect outcomes for participants who received exposure therapy augmentation of antidepressant medication but did confer a benefit for those who preferred and received antipsychotic augmentation.

***Eating disorders.*** Of the two studies targeting disordered eating, only one (Linardon et al., 2023; *N* = 155) utilized a DRPT design, reporting no benefit to offering a choice of approach for binge eating. The second study utilized a PRPT design that evolved part-way through the trial. Given this was our only identified study involving children and adolescents (9–18 years; Loeb et al., 2020; *N* = 59; targeting prodromal Anorexia Nervosa) we report more detail. A strong preference for family-based treatment over individual supportive psychotherapy emerged among carers of these youth, resulting in rates of randomization refusal rising to 75%. A treatment arm was then added for those refusing randomization to receive their treatment of choice; within the design limitations of this study, no impact of offering choice on outcomes was reported.

***Quality of life.*** Three studies utilizing a DRPT design measured this outcome, with two (both targeting depression; Andersson et al., 2023, *N* = 197; Callaghan, Khalil, Morres, & Carter, 2011) finding a benefit to offering patient choice, while one targeting anxiety (Svensson et al., 2021, *N* = 221) did not. Two additional studies with a FRPT design measured quality of life. Koszycki and colleagues (2021 *N* = 97) found no effect of preference matching for a target group with anxiety. We report the effects from Cooper et al. (2018; *N* = 36; targeting depression) under preference strength in the following section.

### Strength of preference

Seven studies (23.3%) measured the strength of preference; six targeted depression (Cooper et al., 2018; Dunlop et al., 2012; Dunlop et al., 2017; Moradveisi, Huibers, Renner, & Arntz, 2014; Raue et al., 2009; Uebelacker et al., 2018) and one targeted anxiety (obsessive-compulsive disorder; Wheaton et al., 2016), with only one study of a DRPT rather than an FRPT design (Raue et al., 2009). Measures varied from categorical (e.g., *mild, moderate*) to continuous (5–100 point scales). At baseline, two studies reported higher preference scores for psychological compared to pharmacological treatment (Raue et al., 2009; Wheaton et al., 2016), and one found no difference (Dunlop et al., 2017). Two studies described the relative effect of preference strength on outcomes. Raue et al. (2009) found preference strength (5-point response scale) was a more sensitive measure of outcome than dichotomous match/mismatch to desired treatment, positively associated with treatment uptake and adherence, but not predictive of depression outcomes. By contrast, Dunlop et al. (2017) found strength of preference (mild, moderate, very strong) did not impact remission rates. When preference was measured separately for treatment approaches, Moradveisi, Huibers, Renner, and Arntz (2014) found preference for one modality was not necessarily negatively correlated with preference for the other approach offered. Cooper et al. (2018) found no impact of preference strength at post-intervention, but at 6-month follow-up, those with higher preference scores who were matched to person-centered counseling (but not to low-intensity CBT) reported a higher quality of life.

**Uptake rates.** Five studies utilized DRPT designs. Of these, three found that offering choice resulted in greater uptake (Brenes et al., 2020, *N* = 500, targeting anxiety; Hegerl et al., 2010, *N* = 368 and Raue et al., 2009, *N* = 60, both targeting depression). Linardon et al. (2023), *N* = 155, binge eating) and Svensson et al. (2021), *N* = 221, anxiety) found no benefit. Two studies utilizing FRPT designs found no benefit of preference matching on uptake (Cooper et al., 2018, *N* = 36, depression; Koszycki, Ilton, Dowell, & Bradwejn, 2022, *N* = 97, anxiety).

### Quality assessment

Supplementary Figure S4 illustrates the frequency of studies meeting CONSORT quality criteria with data for each individual study in Supplementary Table S2. Overall, only 6 studies (20%) were rated as high quality (meeting ≥8 of 10 criteria). Across studies, the top three criteria with the highest compliance were eligibility criteria (86.6%; *n* = 26 studies), baseline demographic and clinical characteristics (73.3%; *n* = 22 studies), and statistical methods used to compare groups for the primary outcome (56.6%; *n* = 17 studies). The criterion with the lowest compliance rate was an explanation of how the sample size was derived (i.e., *a priori* power analyses) with only 8 of 30 studies meeting this criterion (26.6%). The second lowest compliance criterion was the provision of the description of participant losses and exclusions (30%; *n* = 6 studies).

## Discussion

We conducted a systematic review and meta-analysis of the impact of patient choice on treatment outcomes, uptake, and dropout in RCTs across three common and comorbid mental health disorders. Of the 30 studies included at the systematic review stage, where any study design was included, the majority targeted depression (*n* = 23), five focused on anxiety disorders, and two investigated disordered eating. Only one study involved youth (aged 9–18 years), where a strong preference for family-based therapy was driven by carers. All other studies were conducted with middle-aged adults.

Restricting our analyses to the more stringent FRPT and DRPT designs, we identified 22 studies, sufficient to undertake meta-analyses for depression outcomes, and for dropout rates across mixed disorders and within depression studies. This significantly increases our ability to develop robust estimations compared to Delevry and Le (2019). There were insufficient studies to undertake meta-analyses for anxiety or eating disorders, for uptake rates, or using the most stringent choice design alone (DRPT studies) for any outcome.

Across combined FRPT and DRPT study designs, those who were preference-matched or offered choice had greater improvements in depression (*d* = 0.17, *n* = 18). Participants who were not offered a choice or matched to their preferred treatment were 1.46 times more likely to drop out (*n* = 17 studies; mixed target conditions). When dropout was examined purely for depression as a target disorder (*n* = 12 studies), effect sizes were similar but with wider confidence intervals, suggesting a lack of power. Although small, our effects are equivalent in size to the treatment effects found in meta-analyses comparing two established psychotherapies (Lindhiem, Bennett, Trentacosta, & McLear, 2014). These effect sizes are also comparable to those found for other optimization strategies, including personalization of therapy (0.14–0.22; Nye, Delgadillo, & Barkham, 2023) and use of progress feedback (0.15–0.17; de Jong et al., 2021).

A major limitation of this meta-analysis is the small number of robust studies investigating the impact of choice on anxiety and eating disorder outcomes, and in youth samples. It thus remains unclear whether offering choice has the same impact across mental health disorders. For example, anxiety increases the likelihood that ambiguous options will be interpreted negatively, and potential negative outcomes avoided, even at the cost of missing potential gains (Hartley & Phelps, 2012). In contrast, depression is associated with decreased memory of previous rewards to guide decision-making (Rupprechter et al., 2018). Further work on the impact of choice for eating disorders is critical, given the barriers to engaging with treatment. Some first-line treatments for eating disorders already incorporate some collaborative choice of treatment elements (Fairburn and Beglin, 2008; Schmidt, Wade, & Treasure, 2014), and robust testing of choice can inform the improvement of existing therapies.

Only one study investigated the impact of preference on children and adolescents (Loeb et al., 2020), evaluating the choice of carers and not the children. There is an absence of any studies that examine the impact of treatment choice in emerging adults who can be expected to be in treatment independently of family. This somewhat startling omission may indicate some unease about allowing younger people to make such choices. It is clearly a priority for future research, given the escalation of mental health problems in this group, and the subsequent personal and societal costs (McGorry et al., 2024). The explosion of studies examining digital mental health interventions with emerging adults (Taylor et al., 2024) and adolescents (Wüllner et al., 2024) poses a very suitable platform to examine choice and its impact on outcomes.

Further limitations include: (1) only 20% of studies were judged to be high quality; (2) the potential impact of using mixed designs (DRPT and FRPT) to develop estimations. Only seven studies of depression using DRPT were located, 57% of which did not support the advantage of choice; (3) our results do not address impact beyond post-intervention.

In summary, this meta-analysis found that treatment choice in depression can improve outcomes and decrease premature cessation of therapy. Future studies would benefit from incorporating high-quality preference (i.e., DRPT) designs that can best answer questions about treatment choice across depression and other mental health disorders. Greater numbers of DRPT designs would also allow moderator analyses to explore factors that may impact the choice effect: these might include demographic factors (e.g., gender, level of education, socioeconomic status, cultural background), amount and format of treatment information given prior to offering choice (e.g., discussion with a practitioner versus reading a guideline), and whether options for choice matter (e.g., selecting between antidepressant medication and psychotherapy, versus two types of psychotherapy). While there is an indication that a more sensitive measure of choice may be more informative, further work on whether this predicts great variance in outcomes is also required. Also of interest is an investigation of situations where the patient’s choice is at variance with the health professional’s belief in what is needed. Following the lead of depression researchers, anxiety disorders, eating disorders, and youth are key target groups for future research on the benefits of patient choice.

## Supporting information

Johnson et al. supplementary material Johnson et al. supplementary material

## Data Availability

This review has been pre-registered with the Open Science Framework: https://doi.org/10.17605/OSF.IO/SNJXR.

## Supplementary material

The supplementary material for this article can be found at http://doi.org/10.1017/S0033291725000066.
