# Supplementary material for: The impact of patient choice on uptake, adherence, and outcomes across depression, anxiety, and eating disorders: a systematic review and meta-analysis
Source: Psychol Med. 2025 Feb 7;55:e32. doi: 10.1017/S0033291725000066 (PMC12017370; doi:10.1017/S0033291725000066)
Supplement: Johnson et al. supplementary material [file S0033291725000066sup001.docx]

**Supplementary Materials**

**Figure S1**

*Forest plot for Depression outcome: Combined Sample with Fully and Doubly Randomised Trials*


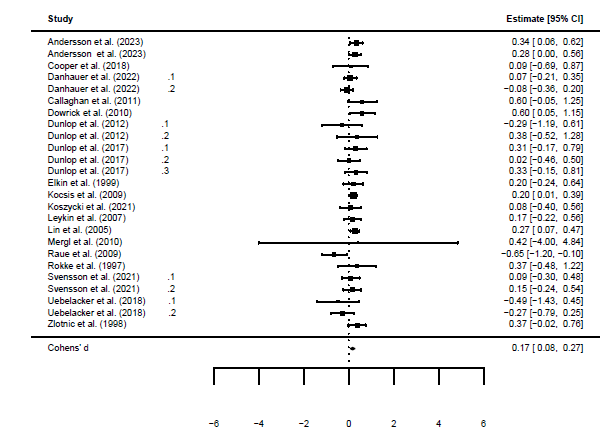


Notes. Andersson et al. (2023) nominated two primary measures for depression 1=Beck Depression Inventory, 2= Patient Health Questionnaire. For studies with multiple comparisons where match is compared to mismatch by therapy: 1=Danhauer et al. (2022) 1=CBT, 2=Yoga; Dunlop et al. (2012) 1=CBT, 2=antidepressant medication (ADM); Dunlop et al. (2017) 1=CBT; 2=Citalopram, 3=Dulox; Svensson et al. (2021) 1=Panic control therapy, 2=Psychodynamic therapy; Uebelacker et al. (2018) 1=Yoga, 2=Healthy living education.

**Figure S2**


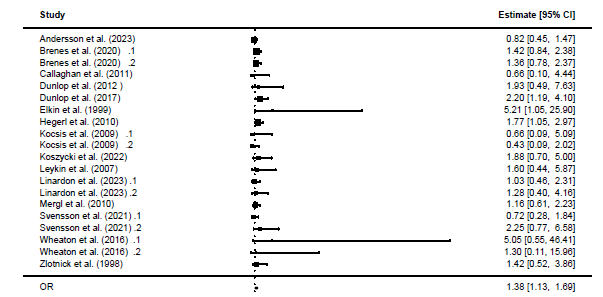
*Forest plot for Dropout Rates: Mixed Target Disorders with Fully and Doubly Randomised Trials*

OR 1.46 [1.17, 1.83]

Note. Kwan et al (2010; OR 16.28 [1.89,140.61]) and Rokke et al. (1997; OR 11.94 [2.37,60.11]) not plotted due to very wide confidence intervals obscuring clarity of plot but are included in OR weighted average. For studies with multiple comparisons where dropout rates are reported by therapy: Brenes et al. (2020) 1=CBT, 2=Yoga; Kocsis et al. (2009) 1= antidepressant medication, 2=CBT; Linardon et al. (2023) 1= multiple mechanisms targeted, 2= single mechanism; Svensson et al. (2021) 1=Panic control therapy, 2=Psychodynamic therapy; Wheaton et al. (2016) 1=Exposure therapy, 2=Risperidone.

**Figure S3**


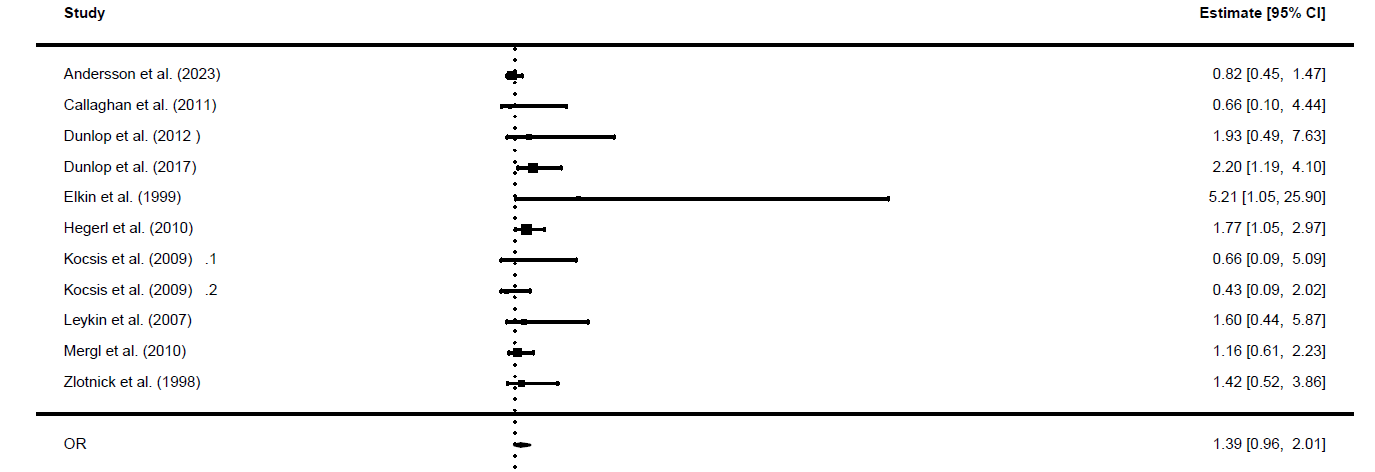
*Forest plot for Dropout Rates: Depression Target with Fully and Doubly Randomised Trials*

OR 1.65 [1.05, 2.59]

Note. Kwan et al (2010; OR 16.28 [1.89,140.61]) and Rokke et al. (1997; OR 11.94 [2.37,60.11]) not plotted due to very wide confidence intervals obscuring clarity of plot but are included in OR weighted average. For studies with multiple comparisons where dropout rates are reported by therapy: Kocsis et al. (2009) 1= antidepressant medication, 2=CBT.

**Figure S4**

*Frequency of Studies Meeting CONSORT Quality Criteria*


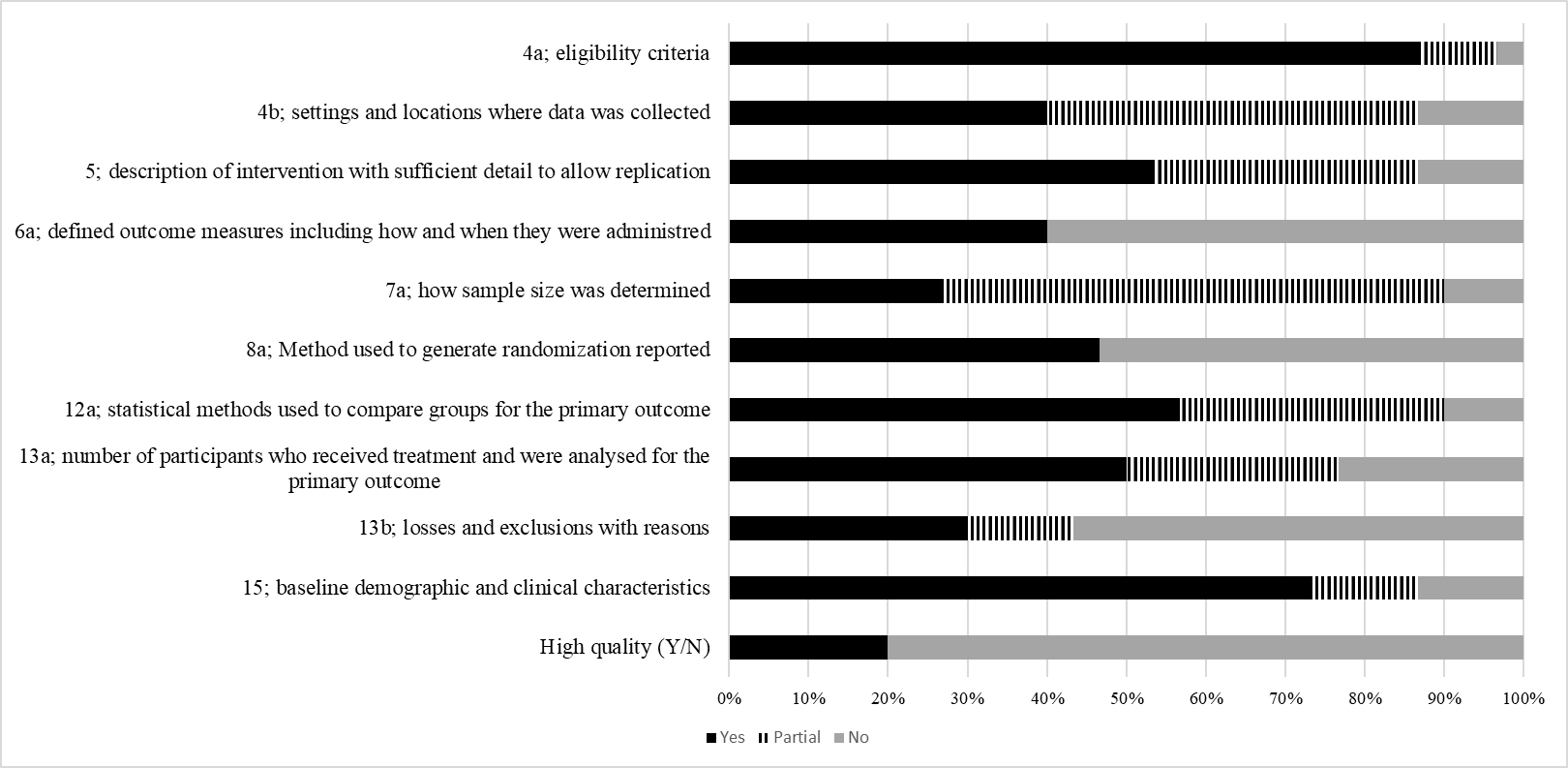


**Table S1.**

*Studies Excluded at Full Text with Reasons*

| Study | Title | DOI | Exclusion reason: |
| --- | --- | --- | --- |
| Bell et al. 2020 | The INternet ThERapy for deprESsion Trial (INTEREST): protocol for a patient-preference, randomised controlled feasibility trial comparing iACT, iCBT and attention control among individuals with comorbid chronic pain and depression. | 10.1136/bmjopen-2019-033350 | Commentary, review, meta-analysis, protocol, secondary analysis |
| Berg et al. 2008 | The relationship of treatment preferences and experiences to outcome in generalized anxiety disorder (GAD) | 10.1348/147608308x297113 | Not a clean comparison between patient choice vs no choice |
| Brenes et al. 2018 | A randomized preference trial of cognitive-behavioral therapy and yoga for the treatment of worry in anxious older adults. | 10.1016/j.conctc.2018.05.002 | Commentary, review, meta-analysis, protocol |
| Brenes et al. 2020 | Corrigendum to ˜A randomized preference trial of cognitive-behavioral therapy and yoga for the treatment of worry in anxious older adults, | 10.1016/j.conctc.2020.100517 | Corrigendum, combined with original study |
| Burke et al. 2006 | PREFER study: a randomized clinical trial testing treatment preference and two dietary options in behavioral weight management--rationale, design and baseline characteristics. | 10.1016/j.cct.2005.08.002 | Target disorder not eating disorders, depression or anxiety |
| Carter et al. 2015 | Preferred intensity exercise for adolescents receiving treatment for depression: a pragmatic randomised controlled trial. | 10.1186/s12888-015-0638-z | No arm offering patient choice vs no choice |
| Carlson et al. 2017 | Protocol for the MATCH study: Mindfulness and Tai Chi for cancer health A preference-based multi-site randomized comparative effectiveness trial (CET) of Mindfulness-Based Cancer Recovery (MBCR) vs. Tai Chi/Qigong (TCQ) for cancer survivors: A preference- | 10.1016/j.cct.2017.05.015 | Commentary, review, meta-analysis, protocol, secondary analysis |
| Chilvers 2001 | Antidepressant drugs and generic counselling for  treatment of major depression in primary care:  randomised trial with patient preference arms | 10.1136/bmj.322.7289.772 | Commentary, review, meta-analysis, protocol, secondary analysis |
|  |  |  |  |
| Dunlop et al. 2014 | "Depression beliefs, treatment preference, and outcomes in a randomized trial for major depressive disorder": Corrigendum. | 10.1016/j.jpsychires.2014.03.001 | Corrigendum, combined with original study |
| Dunn et al. 2002 | The challenge of patient choice and nonadherence to treatment in randomized controlled trials of counseling or psychotherapy. | 10.1207/S15328031US0101_03 | Commentary, review, meta-analysis, protocol, secondary analysis |
| Davidson et al. 2013 | Centralized, stepped, patient preference-based treatment for patients with post-acute coronary syndrome depression: CODIACS vanguard randomized controlled trial. | 10.1001/jamainternmed.2013.915 | Not a clean comparison between patient choice vs no choice |
| Ersner-Hershfield 1979 | Incentive effects of choosing a therapist | 10.1002/1097-4679(197904)35:2 | No mental health measure |
| Gemmell et al. 2011 | The statistical pitfalls of the partially randomized preference design in non-blinded trials of psychological interventions. | 10.1002/mpr.326 | Commentary, review, meta-analysis, protocol, secondary analysis |
| Howard et al. 2006 | Patient preference randomised controlled trials in mental health research | 10.1192/bjp.188.4.303 | Commentary, review, meta-analysis, protocol, secondary analysis |
| Huijbers et al. 2016 | Patients with a preference for medication do equally well in mindfulness-based cognitive therapy for recurrent depression as those preferring mindfulness | 10.1016/j.jad.2016.01.041 | Not a clean comparison between patient choice vs no choice |
| Iacoviello et al. 2007 | Treatment preferences affect the therapeutic alliance: implications for randomized controlled trials. | 10.1037/0022-006X.75.1.194 | No mental health measure |
| Kellett et al. 2023 | Cognitive-behavioural versus cognitive-analytic guided self-help for mild-to-moderate anxiety: a pragmatic, randomised patient preference trial. | 10.1192/bjp.2023.78 | Exploratory study due to very low numbers in no-choice group |
| Kuzminskaite et al. 2021 | Patient Choice in Depression Psychotherapy: Outcomes of Patient-Preferred Therapy Versus Randomly Allocated Therapy. | 10.1176/appi.apt.2020.2020.0042 | Not a clean comparison between patient choice vs no choice; |
| Macias et al. 2005 | Impact of Referral Source and Study Applicants' Preference for Randomly Assigned Service on Research Enrollment, Service Engagement, and Evaluative Outcomes | 10.1176/appi.ajp.162.4.781 | No mental health measure |
| Macias et al. 2009 | Preference in random assignment: Implications for the interpretation of randomized trials | 10.1007/s10488-009-0224-0 | Commentary, review, meta-analysis, protocol, secondary analysis |
| Manthei et al. 1982 | The effect of client choice of therapist on therapy outcome | 10.1007/bf00754338 | No mental health measure |
| Morres 2019 | A pragmatic randomised controlled trial of preferred intensity exercise in depressed adult women in the United Kingdom: secondary analysis of individual variability of depression. | 10.1186/s12889-019-7238-7 | Commentary, review, meta-analysis, protocol, secondary analysis |
| Pynnonen et al. 2018 | Effect of a social intervention of choice vs. control on depressive symptoms, melancholy, feeling of loneliness, and perceived togetherness in older Finnish people: a randomized controlled trial. | 10.1080/13607863.2016.1232367 | No arm offering patient choice vs no choice |
| Sohl et al. 2021 | Ensuring Yoga Intervention Fidelity in a Randomized Preference Trial for the Treatment of Worry in Older Adults. | 10.1089/acm.2020.0476 | Commentary, review, meta-analysis, protocol, secondary analysis |
| Steidtmann et al. 2012 | Patient treatment preference as a predictor of response and attrition in treatment for chronic depression | 10.1002/da.21977 | Not a clean comparison between patient choice vs no choice |
| Tice et al. 2021 | The Selah study protocol of three interventions to manage stress among clergy: a preference-based randomized waitlist control trial. | 10.1186/s13063-021-05845-x | Commentary, review, meta-analysis, protocol, secondary analysis |
| VanDyck et al. 1997 | Does preference for type of treatment matter? A study of exposure in vivo with or without hypnosis in the treatment of panic disorder with agoraphobia | 10.1177/01454455970212003 | Not a clean comparison between patient choice vs no choice |

**Table S2.**

*CONSORT Quality Data for Individual Studies*

|  | 4a | 4b | 5 | 6a | 7a | 8a | 12a | 13a | 13b | 15 |  |
| --- | --- | --- | --- | --- | --- | --- | --- | --- | --- | --- | --- |
|  | Eligibility criteria | Settings and locations where data was collected | Description of intervention with sufficient detail to allow replication | Defined outcome measures including how and when they were administered | How sample size was determined | Method used to generate randomization reported | Statistical methods used to compare groups for the primary outcome | No. of participants who received treatment and were analysed for the primary outcome | Losses and exclusions with reasons | Baseline demographic and clinical characteristics | **High quality**  **Y/N** |
| **Author Name** |  |  |  |  |  |  |  |  |  |  |  |
| Andersson 2023 | Y | Y | Y | Y | N | N | Y | Y | Y | Y | Y |
| Bakker 2000 | N | P | Y | P | N | N | P | P | Y | P | N |
| Bedi 2000 | Y | Y | N | Y | Y | Y | P | N | N | Y | N |
| Brenes 2020/Danhauer 2022 | Y | Y | P | Y | Y | Y | P | Y | P | Y | N |
| Callaghan 2011 | Y | P | N | P | Y | Y | N | Y | Y | Y | N |
| Cooper 2018 | Y | P | P | P | P | Y | P | P | P | P | N |
| Dowrick 2011 | Y | P | N | P | N | N | P | P | N | Y | N |
| Dunlop 2012 | Y | P | Y | P | N | N | P | Y | N | Y | N |
| Dunlop 2017 | Y | P | Y | Y | N | Y | Y | P | N | Y | N |
| Elkin 1999 | Y | P | Y | P | N | N | N | Y | Y | N | N |
| Gum 2006 | Y | N | P | P | N | N | Y | Y | N | Y | N |
| Hegerl 2010 | Y | Y | Y | P | Y | Y | Y | Y | Y | Y | Y |
| Kocsis 2009 | Y | N | P | P | N | N | Y | Y | N | Y | N |
| Koszycki 2022 | Y | N | Y | Y | N | Y | Y | P | N | Y | N |
| Kwan 2010 | P | P | Y | P | N | Y | Y | N | N | N | N |
| Leuzinger-Bohleber 2019 | Y | P | Y | P | Y | Y | Y | Y | N | Y | Y |
| Leykin 2007 | Y | Y | N | P | P | N | P | P | N | Y | N |
| Lin 2005 | Y | Y | Y | P | N | N | P | P | N | Y | N |
| Linardon 2023 | Y | P | Y | Y | N | Y | Y | Y | Y | Y | Y |
| Loeb 2020 | Y | P | Y | Y | N | Y | Y | Y | Y | Y | Y |
| Mergl 2010 | Y | P | P | Y | Y | N | Y | P | P | Y | N |
| Moradveisi 2014 | Y | Y | P | Y | N | N | P | N | N | N | N |
| Raue 2009 | Y | Y | Y | P | P | N | Y | Y | P | Y | N |
| Rokke 1997 | Y | Y | Y | Y | N | N | P | N | N | P | N |
| Svensson 2021 | Y | Y | Y | Y | Y | Y | Y | Y | Y | Y | Y |
| Uebelacker 2018 | Y | P | Y | Y | N | N | Y | N | N | Y | N |
| Van 2009 | Y | Y | P | P | N | Y | Y | Y | N | Y | N |
| Ward 2000 | Y | Y | P | P | Y | Y | Y | Y | Y | P | N |
| Wheaton 2016 | P | P | P | P | N | N | Y | N | N | N | N |
| Zlotnick 1998 | P | N | N | P | N | N | N | N | N | Y | N |
